# Antibiotic Prescribing Practices and Clinical Outcomes of Pediatric Patients with *Campylobacter* Enterocolitis

**DOI:** 10.3390/children10010040

**Published:** 2022-12-25

**Authors:** Dasom Wi, Soo-Han Choi

**Affiliations:** 1 Department of Pediatrics, Hallym University Dongtan Sacred Heart Hospital, Hwaseong 18450, Republic of Korea; 2 Biomedical Research Institute, Pusan National University Hospital, Busan 49241, Republic of Korea; 3 Department of Pediatrics, School of Medicine, Pusan National University, Busan 49241, Republic of Korea

**Keywords:** *Campylobacter*, enterocolitis, anti-bacterial agents, treatment outcome, child

## Abstract

Antibiotics are not routinely recommended for patients with *Campylobacter* enterocolitis. We conducted a retrospective review of hospitalized patients younger than 18 years diagnosed with *Campylobacter* enterocolitis from July 2015 to December 2019. This study aimed to investigate antibiotic prescribing practices and the clinical outcomes and to evaluate the factors associated with antibiotic use. A total of 157 patients (median age, 10.7 years) were included in this study. Most patients (93.0%) had a fever, and a quarter of the patients complained of bloody diarrhea. The overall antibiotic prescribing rate was 36.7% (57/157), 91.2% of the patients received antibiotics within hospital day 2. The semi-annual antibiotic prescribing rate ranged from 16.7 to 50.0%. There were no increasing or decreasing trends in antibiotic prescribing rates. Cephalosporins were the most prescribed antibiotics for initial antibiotic therapy. Azithromycin use increased significantly during the study period. The independent factors associated with early antibiotic therapy were leukocytosis (adjusted odds ratio (aOR), 3.95; 95% confidence interval (CI), 1.76–9.27), C-reactive protein ≥50 mg/L (aOR, 4.19; 95% CI, 1.84–10.21), and performing abdominal imaging studies (aOR, 3.44; 95% CI, 1.55–7.99). There was no significance in defervescence between the early and no-antibiotic therapy groups (*p* = 0.232). A careful assessment of the need for antibiotic therapy in patients with acute diarrhea should be conducted to avoid unnecessary use. After identifying the causative pathogens, the appropriateness of antibiotic prescription should be evaluated.

## 1. Introduction

*Campylobacter* is one of the most common causes of bacterial enterocolitis in humans, particularly children and young adults [1,2,3,4]. The World Health Organization (WHO) noted that *Campylobacter* is 1 of 4 key global causes of diarrheal diseases [5]. Active surveillance through the Foodborne Diseases Active Surveillance Network (FoodNet) indicated that about 20 cases per 100,000 people are diagnosed each year in the United States (US). The Centers for Disease Control and Prevention (CDC) estimated that 1.5 million people in the US become ill from *Campylobacter* infection every year [6]. *Campylobacter jejuni* (*C. jejuni*) and *Campylobacter coli* (*C. coli*) account for most human infections [2]. *Campylobacter* enterocolitis is characterized by fever, diarrhea, and abdominal cramps, and is often accompanied by bloody stools [7,8,9,10]. Most bacterial enterocolitis, including *Campylobacter* infection, is self-limited and resolves without specific antimicrobial treatment [1,11].

Antibiotics are not routinely recommended for most patients with suspected or proven bacterial enterocolitis [1,11,12,13]. Antibiotic therapy is considered for patients aged younger than 3 months, with suspected sepsis or disseminated infection, severe disease, immune dysfunction, or other comorbidities associated with complications [1,11]. Antimicrobial treatment should be modified or discontinued when a clinically plausible organism is identified. Antimicrobial susceptibility testing should be considered when a therapeutic agent is selected [13]. *Campylobacter* species have inherited resistance to trimethoprim and beta-lactam antibiotics including penicillins and cephalosporins [1]. The recommended antibiotics is azithromycin in pediatric patients with uncomplicated *Campylobacter* infections who merit antibiotic therapy [1,13].

Data on antibiotic prescribing practices in pediatric patients with *Campylobacter* enterocolitis are limited [9,10]. Understanding the pattern of antibiotic use for *Campylobacter* infections could inform antimicrobial stewardship efforts. This study aimed to investigate antibiotic prescribing practices for pediatric patients diagnosed with *Campylobacter* enterocolitis and to evaluate the factors associated with antibiotic use and the clinical outcomes.

## 2. Materials and Methods

### 2.1. Study Patients and Definition

We conducted a retrospective study of hospitalized pediatric patients (younger than 18 years old) with acute enterocolitis caused by *Campylobacter* at Hallym University Dongtan Sacred Heart Hospital, the Republic of Korea, from July 2015 to December 2019. The inclusion criteria of this study were as follows: (1) patients who were hospitalized due to acute symptoms compatible with infectious enterocolitis such as fever, abdominal pain, diarrhea, or vomiting within seven days of onset, and (2) patients in whom *Campylobacter* was confirmed by the multiplex polymerase chain reaction (PCR) assay (Seeplex^®^ Diarrhea-B2 ACE Detection, Seegene Inc., Seoul, Republic of Korea) of stool specimens. The *Campylobacter* species detectable by the multiplex PCR assay were *C. jejuni* and *C. coli*. We excluded patients with chronic medical diseases or *Campylobacter* co-infection with other organisms from the study.

We defined patients who received antibiotic therapy within the second day of hospitalization as the early antibiotic group. Patients who did not receive antibiotic therapy during hospitalization and after discharge were classified as the no-antibiotic group. Referring to the 2017 Infectious Diseases Society of America (IDSA) guidelines [13], we defined appropriate antibiotic therapy as prescribing azithromycin or discontinuing antibiotics other than azithromycin after the patient was confirmed to have *Campylobacter* enterocolitis. Inappropriate antibiotic therapy was defined as maintaining or initiating antibiotics other than azithromycin after confirming the result.

### 2.2. Data Collection and Analysis

A review of medical records was performed to collect clinical information. We collected data on demographics, the onset time of fever, and gastrointestinal symptoms at hospital admission. Laboratory data on peripheral blood white blood cell (WBC) counts and C-reactive protein (CRP), the hospital day of stool PCR result reporting, and data on imaging studies, such as abdominal ultrasonography (US) or computer tomography (CT), were collected. We investigated treatments for *Campylobacter* enterocolitis, the type of antibiotics prescribed, and the clinical outcomes of the study patients. The clinical outcome variables were fever defervescence by hospital day and length of hospital stay (LOS). The defervescence day was defined as the hospital day when the last fever occurred. We analyzed the factors associated with early antibiotic therapy and compared clinical outcomes between early and no-antibiotic groups.

### 2.3. Statistical Analysis

We used descriptive statistics, including medians, interquartile ranges (IQRs), counts, and rates or proportions with 95% confidence intervals (95% CIs). In comparisons between the two groups, Fisher’s exact test and the Mann–Whitney U test were used for categorical and continuous variables, respectively. The Chi-square test for trend was used to evaluate trends of antibiotic prescribing rates over time. The correlation between positive rate of *Campylobacter* and antibiotic prescribing rate was evaluated using Spearman’s correlation analysis. Fever defervescence curves according to hospital day between the early and no-antibiotics groups were compared by the log-rank test. Multiple logistic regression analysis was performed to assess the independent factors associated with the clinical decision for early antibiotic therapy, and adjusted odds ratios (aORs) and 95% CIs were calculated. We chose potential predictors using statistically significant variables in the univariable analysis. We tested for multicollinearity with variance inflation factors to control for confounding variables and performed the Hosmer–Lemeshow test for goodness-of-fit. Two-sided *p*-values of < 0.05 were considered statistically significant. The data were analyzed using Prism 9.4.1 (GraphPad Software Inc., San Diego, CA, USA).

## 3. Results

### 3.1. Patients Characteristics

**Figure 1:**
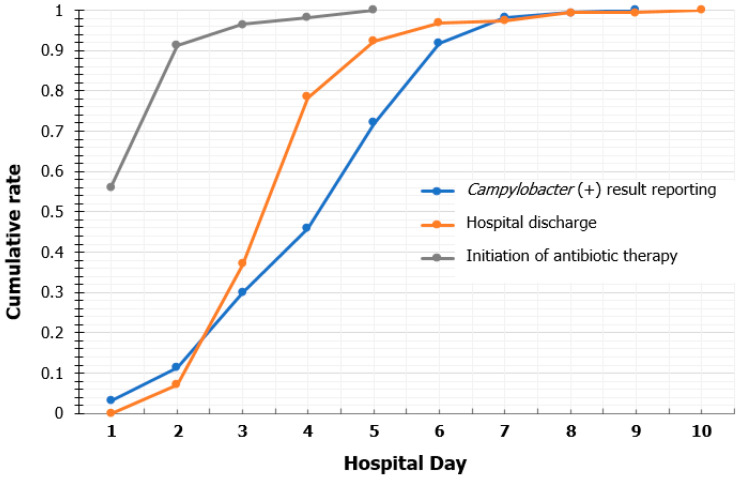
Cumulative rate of *Campylobacter* enterocolitis confirmations, discharge, and the initiation of antibiotic therapy according to hospital day.

A total of 157 patients confirmed with *Campylobacter* enterocolitis were eligible for this study. The median monthly number of patients was 2 (range, 0–17; IQR, 1–4). The monthly positive PCR rate for *Campylobacter* ranged from 0% to 38.6% (median, 6.9%; 95% CI, 4.2–7.4%). *Campylobacter* infection was observed through the years and peaked in the summer season (Figure S1). The characteristics of the study patients are summarized in Table 1. The median age of the patients was 10.7 years (IQR, 6.3–14.4 years). Most patients (93.0%) had a fever before or at admission and the median fever duration was 3 days (IQR, 2–4 days). A quarter of the patients complained of bloody diarrhea at admission, and 61.8% of the total patients tested positive for stool occult blood. In the initial laboratory findings, the proportion of patients with WBC counts ≥12,000/mm^3^ and CRP level of ≥ 50 mg/L were 33.8% (53/157) and 57.3% (90/157), respectively. Among 71 patients who underwent abdominal US or CT, 97.2% (69/71) had bowel wall thickening or dilatation, mesenteric lymphadenopathy, or abnormal fluid collection in the intraabdominal cavity. The median time from admission to stool PCR result reporting was 5 days (IQR, 3–6 days), and 29.3% (46/157) were confirmed to have *Campylobacter* infection before discharge (Figure 1).

### 3.2. Antibiotic Prescribing Practices

**Figure 2:**
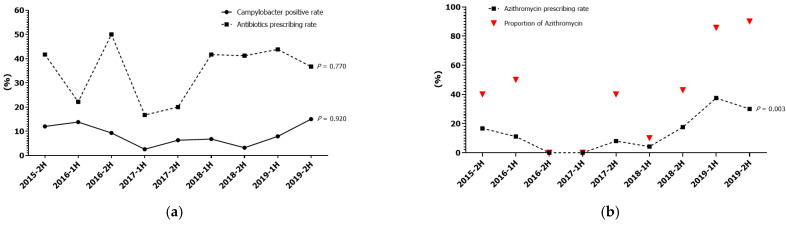
The trends in *Campylobacter* positive rates and antibiotic prescribing rates (**a**); the use of azithromycin (**b**).

During the study period, the overall antibiotics prescribing rate was 36.7% (57/157). The semi-annual antibiotic prescribing rate ranged from 16.7 to 50.0% (median, 41.2%; 95% CI, 20.0–43.8%). There were no increasing or decreasing trends in antibiotic prescribing rates (Figure 2a). There was no correlation between the positive rate of *Campylobacter* in stool PCR and the antibiotic prescribing rate (Spearman’s rho = 0.293; *p* = 0.442). Among 57 patients receiving antibiotic therapy, 91.2% (52/57) were included in the early antibiotic group (32 on hospital day 1 and 20 on hospital day 2) and 33.3% (19/57) were confirmed with *Campylobacter* infection before hospital discharge. Six patients (10.5%) received antibiotics after stool PCR result reporting. Of these, four were confirmed with *Campylobacter* infection within hospital day 2.

Cephalosporins were the most prescribed antibiotics for initial antibiotic therapy (Table 2). Twenty-four (42.1%, 24/57) patients received azithromycin as combination therapy or alone. The trend in azithromycin use increased significantly during the overall study period (Figure 2b).

Of 19 patients confirmed with Campylobacter enterocolitis before discharge, eight (42.1%) were treated with azithromycin as empirical or targeted therapy, and antibiotic therapy was discontinued in four (21.1%) after confirming the stool PCR results. However, 36.8% of the patients received inappropriate antibiotic therapy even after confirming *Campylobacter* infection.

A comparison of the characteristics between the early and no-antibiotic groups is described in Table 3. The proportion of patients with fever on hospital day 1 or 2 (90.4 vs. 74.0%; *p* = 0.019) and abdominal imaging studies at admission (63.5 vs. 35.0%; *p* = 0.001) were significantly higher in the early antibiotic group than in the no-antibiotic group. In the early antibiotic group, there were fewer patients with bloody diarrhea than in the no-antibiotic group (11.5 vs. 32.0%; *p* = 0.006). Compared to the no-antibiotic group, WBC counts (median, 12,550 vs. 9150/mm^3^; *p* = 0.001) and CRP (median, 93.2 vs. 46.2 mg/L; *p* < 0.001) values were significantly higher in the early antibiotic group.

The presence of bloody diarrhea, fever on hospital day 1 or 2, performing abdominal imaging studies at admission, and WBC and CRP values were determined to be potential predictors associated with early antibiotic therapy. In the multiple logistic regression analysis (Table 4), leukocytosis (WBC ≥ 12,000/mm^3^), high CRP values (≥50 mg/L), and performing abdominal imaging studies at admission were independent factors associated with early antibiotic therapy.

### 3.3. Clinical Outcomes

**Figure 3:**
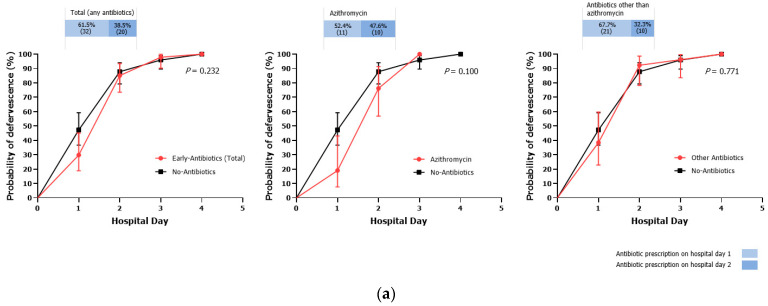

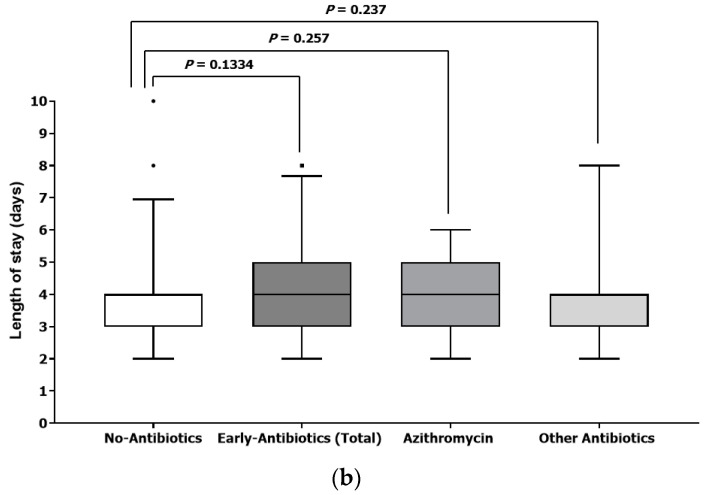
Comparison of fever defervescence curves (**a**) and length of stay (**b**) between the early and no antibiotic groups. The error bar in (**a**) indicates 95% confidence intervals. The box plot in (**b**) shows median and interquartile range. The error bar in (**b**) indicates 2.5–97.5 percentile.

Of 125 patients with fever during hospitalization, the rate of fever defervescence by hospital day 3 was 96.8%. There were no significant differences in defervescence by hospital day 3 between the early and no-antibiotic groups: early (any antibiotics) vs. no-antibiotic, 97.9 vs. 95.9%; azithromycin vs. no-antibiotic, 100.0 vs. 95.9%; antibiotics other than azithromycin vs. no-antibiotic, 96.1 vs. 95.9% (all *p*-values > 0.999). There was no statistical difference in defervescence curves between the three groups (Figure 3a). The median LOS was 4 days (range, 2–10; IQR, 3–4) in all patients, and there was no difference between the early antibiotic groups and no-antibiotic group (Figure 3b). Among all study patients, no patients had concomitant *Campylobacter* bacteremia or needed intensive care unit admission.

## 4. Discussion

In our study of 157 patients confirmed with *Campylobacter* enterocolitis, the overall antibiotic prescribing rate was 36.7%. There were no increasing or decreasing trends in antibiotic prescribing rates over time, but the prescription rate of azithromycin increased significantly during the overall study period. Antibiotics therapy was initiated before confirming *Campylobacter* infections in most patients, and more than 90% of the patients received antibiotic therapy within the second day of hospitalization. Leukocytosis (WBC ≥ 12,000/mm^3^), high CRP values (≥50 mg/L), and performing abdominal imaging studies at admission were independent factors associated with early antibiotic therapy. However, there were no differences in fever defervescence and the duration of hospitalization between patients with early antibiotic therapy and those with no-antibiotic therapy.

In this study, the semi-annual positive PCR rate of *Campylobacter* in stool samples ranged from 2.6 to 15.0%. According to data from the sentinel surveillance of acute infectious diarrhea by the Korea Disease Control and Prevention Agency, bacterial pathogens were isolated from 11.5–23.7% of the samples between 2012 and 2016 [12,14]. *Campylobacter* was one of the most frequently isolated bacterial pathogens, along with nontyphoidal *Salmonella* (NTS) and *Clostridium perfringens*, through the sentinel surveillance of acute infectious diarrhea in Korea from 2015 to 2019 [14].

Since bacterial causes of diarrhea may have similar clinical presentations, it is challenging to distinguish *Campylobacter* infections from enterocolitis caused by other organisms without microbiologic testing. It is not always necessary to determine the causative organism of diarrhea. However, diagnostic tests to determine the cause may be needed for patients at a high risk of severe illness and for whom the identification of pathogens would be important or for public health [13]. The 2017 IDSA guidelines recommend that stool testing should be performed for *Salmonella*, *Shigella*, *Campylobacter*, *Yersinia*, *Clostridioides difficile*, and Shiga toxin producing *Escherichia coli* (STEC) in people with diarrhea accompanied by fever, bloody or mucoid stools, severe abdominal cramping or tenderness, or signs of sepsis [13]. In a Taiwan study comparing the clinical and laboratory features of NTS and *Campylobacter jejuni* enterocolitis in children, aged ≥ 5 years, leukocytosis (≥ 10,000/mm^3^, abdominal pain, and watery diarrhea were identified as good predictors of *Campylobacter* enterocolitis [15]. Bae et al. [8] noted that pediatric patients with *Campylobacter* enterocolitis were significantly older and had higher CRP levels than those with *Salmonella* or *Clostridium perfringens* infections. The predictors of *Campylobacter* enterocolitis were aged ≥ 103.5 months and CRP ≥ 4.55 mg/dL in Bae et al.’s study. Although this study did not perform a comparative analysis between *Campylobacter* and other bacterial pathogens, older age, abdominal pain, leukocytosis, and high CRP levels were characteristic features in our study. However, when high CRP levels and significant leukocytosis are observed in patients with acute enterocolitis, clinicians may prescribe antibiotics due to concerns about serious illness. In our study, leukocytosis (WBC ≥ 12,000/mm^3^), high CRP values (≥50 mg/L), and performing abdominal imaging studies were independent factors associated with the use of antibiotics in the early days of hospitalization. In an Australian study, Moffatt et al. [16] reported that one-third of *Campylobacter*-associated hospitalized patients received antibiotics, either empirically or as targeted therapy, for confirmed campylobacteriosis. Vomiting, electrolyte imbalance, and obtaining blood cultures were included in factors associated with antibiotic treatment in the study.

Most gastrointestinal infections are self-limited among healthy people. Antibiotics are usually not recommended for patients with suspected or proven bacterial enterocolitis. Unnecessary antibiotic use contributes to antibiotic resistance, increase in prolonged *Salmonella* shedding and the occasional shedding of antibiotic-resistant *Campylobacter*, and may increase the risk of hemolytic uremic syndrome in patients with STEC infection [11,17]. Antibiotics were prescribed in 13% of acute gastroenteritis visits in the US nationwide during 2006–2015. Antibiotic prescribing for *Campylobacter* and NST was 44.8% and 31.7%, respectively [18]. During 2013–2018, antimicrobial drugs were prescribed for 6.8% of the patients with acute gastroenteritis in general practice in Australia, including 35.7% of *Salmonella* and 54.1% of *Campylobacter* infections [19]. In a Korean study using National Health Insurance Service claims data from 2016 to 2017, the antibiotic prescribing rate for adults with acute infectious diarrhea was 46.7%, suggesting that unnecessary antibiotics were used often [20]. In another Korean study of a multicenter one-day point prevalence survey in 2018, 25.2% of the antibiotic prescriptions for infectious colitis were inappropriate [21]. If empirical antibiotic therapy is indicated for patients with acute diarrhea, antibiotic prescription should be modified or discontinued after identifying the causative pathogen. The assessment of appropriateness was limited in our study because two-thirds of the patients were confirmed with *Campylobacter* infection after discharge. However, 36.8% of the prescriptions among patients confirmed with *Campylobacter* before discharge were inappropriate.

A meta-analysis of 11 randomized controlled trials on the effects of antibiotic treatment on the duration of symptoms caused by *Campylobacter* infections in children and adults showed that antibiotic treatment shortened the duration of intestinal symptoms by 1.32 days [22]. A prospective randomized assessor-blinded study showed that the administration of a single oral dose of 30 mg/kg of azithromycin early after disease onset effectively eradicated *Campylobacter* and accelerated clinical cures in pediatric *Campylobacter* enterocolitis [23]. Our study did not show a shortened duration of fever defervescence or hospitalization among patients who received azithromycin. This finding may be associated with the fact that antibiotics were prescribed to patients who had more severe clinical features.

Our study had several limitations. First, *Campylobacter* enterocolitis was confirmed by panel-based multiplex PCR assay. Stool cultures for *Campylobacter* were not performed in our study patients. Data for antibiotic susceptibility testing were not available. Multiplex PCR assay can simultaneously detect several enteric pathogens and *Campylobacter* may not be the cause of enterocolitis at present [24,25]. However, this study excluded patients with multiple organisms other than *Campylobacter*. Second, the appropriateness of antibiotic prescribing could be evaluated in only a few patients. Despite this, our study provided comprehensive data on antibiotic prescribing practices in pediatric patients suspected of bacterial enterocolitis.

## 5. Conclusions

Although some pediatric patients with *Campylobacter* enterocolitis had severe clinical features, the benefits of antibiotic therapy were not apparent in this study. Even after confirming *Campylobacter* infections, inappropriate antibiotic use was frequently observed. A careful assessment of the need for empirical or targeted antibiotic treatment in patients with acute diarrhea should be performed to avoid unnecessary antibiotic use. Moreover, after identifying the causative pathogens, the appropriateness of antibiotic prescription should be evaluated.

## Figures and Tables

**Table 1:** Characteristics of the study patients.

Characteristics	Total N = 157	Age < 7 Years N = 45	Age ≥ 7 Years N = 112	*p*-Value
Sex, male (%)	94 (59.9)	18 (40.0)	76 (67.9)	0.002
Age, years, median (range)	10.7 (0.3–17.5)	4.1 (0.3–6.8)	13.0 (7.0–17.5)	NA
Occurrence in summer season, Jun ~ Aug (%)	85 (54.1)	27 (60.0)	58 (51.8)	0.380
Clinical manifestation before or at admission				
Fever (%)	146 (93.0)	43 (95.6)	103 (92.0)	0.730
Fever duration, days, median (range)	3 (1–7)	2 (1–6)	3 (1–7)	0.328
Abdominal pain (%)	134 (85.4)	32 (71.1)	102 (91.1)	0.002
Bloody diarrhea (%)	39 (24.8)	22 (48.9)	17 (15.2)	<0.001
Fever during hospitalization	125 (79.6)	35 (77.8)	90 (80.4)	0.827
Initial laboratory findings				
White blood cell count (/mm^3^), median (range)	10,100 (3300–22,400)	9600 (4200–22,400)	10,200 (3300–20,200)	0.958
Absolute neutrophil count (/mm^3^), median (range)	7811 (250–20,518)	7228 (250–20,518)	8057 (2225–17,756)	0.267
C-reactive protein (mg/L), median (range)	58.4 (1–211.3)	48.3 (1–142.8)	60.9 (2.1–211.3)	0.073
Positive for stool white blood cell (%)	75 (47.8)	20 (44.4)	55 (49.1)	0.724
Positive for stool occult blood (%)	97 (61.8)	28 (62.2)	69 (61.6)	>0.999
Abdominal CT or ultrasonography performed (%)	71 (45.2)	14 (31.1)	57 (50.9)	0.033
Abnormal findings in imaging study ^1^ (%)	69/71 (97.2)	14/14 (100.0)	55/57 (96.5)	>0.999
Bowel involvement in imaging study				NA
Ileum (%)	35/71 (49.3)	7/14 (50.0)	28/57 (49.1)	
Cecum (%)	23/71 (32.4)	6/14 (42.9)	17/57 (29.8)	
Ascending colon (%)	20/71 (28.2)	4/14 (28.6)	16/57 (28.1)	
Transverse colon (%)	10/71 (14.1)	1/14 (7.1)	9/57 (15.8)	
Descending colon (%)	6/71 (8.4)	1/14 (7.1)	5/57 (8.8)	
Sigmoid colon/Rectum (%)	11/71 (15.5)	1/14 (7.1)	10/57 (17.5)	
Whole colon (%)	21/71 (29.6)	4/14 (28.6)	17/57 (29.8)	

^1^ Abnormal bowel wall thickening or dilatation, lymphadenopathy, or fluid collection in the intraabdominal cavity; CT, computed tomography; NA, non-applicable.

**Table 2:** Antibiotic prescribing patterns.

Antibiotic Prescribing Patterns	N = 57
Initial antibiotic therapy (%)	
Penicillins	8 (14.0)
Cephalosporins	24 (42.1)
Azithromycin	17 (29.8)
Azithromycin + Penicillins	2 (3.5)
Azithromycin + Cephalosporins	5 (8.8)
Others	1 (1.8)
Early antibiotic group ^1^	52 (91.2)
With azithromycin ^2^	21/52 (40.4)
Without azithromycin	31/52 (59.6)
Patients confirmed with *Campylobacter* (+) before discharge (%)	19 (33.3)
Empirical therapy with azithromycin	3/19 (15.8)
Targeted therapy with azithromycin after confirming the result	5/19 (26.3)
Stop antibiotics other than azithromycin after confirming the result	4/19 (21.1)
Maintenance or initiation of antibiotics other than azithromycin after confirming the result	7/19 (36.8)

^1^ Initiation of antibiotics therapy within the second day of hospitalization. ^2^ Included combination therapy of azithromycin and other antibiotics.

**Table 3:** Comparison between the early and no-antibiotic groups.

Variables	Early Antibiotic Group ^1^ (N = 52)	No-Antibiotic Group ^2^ (N = 100)	*p*-Value
Age, years, median (range)	12.7 (0.3–17.5)	10.3 (1.2–17.0)	0.109
Summer season, Jun–Aug (%)	27 (51.9)	56 (56.0)	0.732
Clinical manifestations before or at admission			
Fever (%)	52 (100.0)	90 (90.0)	0.016
Duration of fever, days, median (IQR)	3 (2-3)	3 (2–4)	0.897
Abdominal pain (%)	47 (90.4)	84 (84.0)	0.330
Bloody stool (%)	6 (11.5)	32 (32.0)	0.006
Fever on hospital day 1 or 2	47 (90.4)	74 (74.0)	0.019
Laboratory findings on admission			
White blood cell count (/mm^3^), median (range)	12,550 (4900–20,200)	9150 (4200–22,400)	0.001
C-reactive protein (mg/L), median (range)	93.2 (12.1–196.1)	46.2 (1.0–211.3)	<0.001
Imaging studies performed at admission (%)	33 (63.5)	35 (35.0)	0.001
Abnormal findings in imaging studies (%)	31/33 (93.9)	35/35 (100.0)	0.232
Days from admission to result reporting, median (range)	5 (1–7)	5 (1–9)	0.471

^1^ Initiation of antibiotics therapy within the second day of hospitalization. ^2^ No antibiotic therapy during hospitalization. IQR, interquartile range.

**Table 4:** Factors associated with early antibiotic therapy.

Variables	Univariable Analysis		Multiple Logistic Analysis	
	Odds Ratio (95% CI)	*p*-Value	Odds Ratio (95% CI)	*p*-Value
Bloody diarrhea	0.28 (0.11–0.72)	0.006	0.36 (0.12–0.97)	0.054
Fever on hospital day 1 or 2	3.30 (1.23–8.31)	0.019	1.64 (0.60–4.79)	0.344
PB WBCs ≥ 12,000/mm^3^	3.69 (1.82–7.31)	<0.001	3.95 (1.76–9.27)	0.001
CRP ≥ 50 mg/L	4.56 (2.10–9.67)	<0.001	4.19 (1.84–10.21)	0.001
Imaging study performed	3.23 (1.64–6.27)	0.001	3.44 (1.55–7.99)	0.003

CI, confidence interval; PB WBCs, peripheral blood white blood cells; CRP, C-reactive protein.

## Data Availability

Data supporting the results of this study are available from the corresponding author on reasonable request.

## Supplementary Materials

The following supporting information can be downloaded at: https://www.mdpi.com/article/10.3390/children10010040/s1, Figure S1: Monthly distribution of study patients and positive rate of *Campylobacter* species by multiplex stool polymerase chain reaction assay.
