# Supplementary figures and images for: Antibiotic Prescribing Practices and Clinical Outcomes of Pediatric Patients with Campylobacter Enterocolitis
Source: Children (Basel). 2022 Dec 25;10(1):40. doi: 10.3390/children10010040 (PMC9856514; doi:10.3390/children10010040)

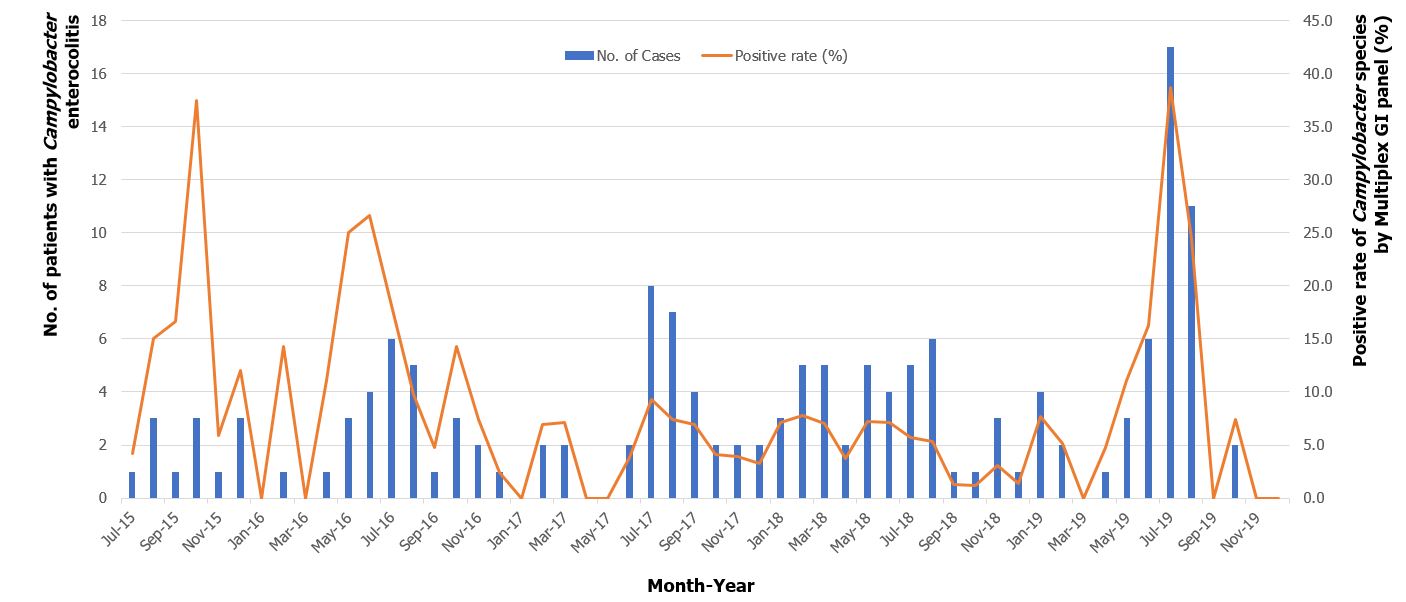

Supplement: Supplementary file 1 [file children-10-00040-s001.zip › Supplementary Materials/Figure S1.JPG]
